# Move faster, think later: Women who play action video games have quicker visually-guided responses with later onset visuomotor-related brain activity

**DOI:** 10.1371/journal.pone.0189110

**Published:** 2018-01-24

**Authors:** Diana J. Gorbet, Lauren E. Sergio

**Affiliations:** 1 School of Kinesiology and Health Science, York University, Toronto, Ontario, Canada; 2 Centre for Vision Research, York University, Toronto, Ontario, Canada; IRCCS E. Medea, ITALY

## Abstract

A history of action video game (AVG) playing is associated with improvements in several visuospatial and attention-related skills and these improvements may be transferable to unrelated tasks. These facts make video games a potential medium for skill-training and rehabilitation. However, examinations of the neural correlates underlying these observations are almost non-existent in the visuomotor system. Further, the vast majority of studies on the effects of a history of AVG play have been done using almost exclusively male participants. Therefore, to begin to fill these gaps in the literature, we present findings from two experiments. In the first, we use functional MRI to examine brain activity in experienced, female AVG players during visually-guided reaching. In the second, we examine the kinematics of visually-guided reaching in this population. Imaging data demonstrate that relative to women who do not play, AVG players have less motor-related preparatory activity in the cuneus, middle occipital gyrus, and cerebellum. This decrease is correlated with estimates of time spent playing. Further, these correlations are strongest during the performance of a visuomotor mapping that spatially dissociates eye and arm movements. However, further examinations of the full time-course of visuomotor-related activity in the AVG players revealed that the decreased activity during motor preparation likely results from a later onset of activity in AVG players, which occurs closer to beginning motor execution relative to the non-playing group. Further, the data presented here suggest that this later onset of preparatory activity represents greater neural efficiency that is associated with faster visually-guided responses.

## Introduction

Action video games (AVGs) require focused attention, quick processing of sensory information, and rapid responses. A large body of research suggests that AVG play increases proficiency in a wide range of visuospatial and attentional processing skills with a concurrent reduction in movement reaction time (for reviews, see [1–4]. Importantly, some of these improvements may be transferable to unrelated tasks [2,5–10]. This transfer of skill proficiency may result from more efficient use of sensory input to quickly learn and perform new tasks requiring skills similar to those improved by gaming [11]. The potential generalizability of skill enhancement makes AVGs a highly attractive potential tool for skill training and neurorehabilitation approaches.

Some of what we know about the effects of action video games on the brain comes from training paradigm studies in which individuals with little or no prior AVG exposure are tested using various measures and then play a prescribed amount of a specific game, usually over a period of several weeks, prior to re-testing. AVG training studies have provided evidence that playing this genre of game can improve skills including visual resolution [12] contrast sensitivity [13], visuospatial processing [14–16], and visuospatial attention [17–19]. However, improvements in these domains have not been confirmed by all studies [16,20,21], suggesting that training paradigms, or individual characteristics of participants may influence the outcome of training. Training studies examining the neural correlates of these acquired skill improvements have demonstrated decreased activity in regions of the frontal, parietal, and occipital cortex [22,23] as well as sex-dependent changes in activity and connectivity of brain regions associated with reward such as the orbitofrontal cortex, amygdala and nucleus accumbens [24].

In addition to research examining the immediate effects of relatively brief training, there are also several studies of the behavioural correlates of AVG play in experienced players (“gamers”) who have played these games often and consistently over numerous years. These studies confirm that the changes to visuospatial perception [25–28], attention-related skills [18,29–32], reductions in reaction time [33,34], and enhanced multisensory processing [35] are also observed in players with extensive AVG experience that has occurred outside of a laboratory setting. Far fewer studies have examined the underlying neural correlates of a history of extensive AVG play. Bavelier and colleagues [36] demonstrated decreased activity within frontal and parietal regions during a visual search task with evidence of greater moving distractor suppression in the middle temporal cortex, suggesting a potential neural basis underlying improved spatial attention in gamers. In addition, amounts of AVG experience positively correlate with grey matter volume within the parahippocampal region, the occipital cortex, and the posterior parietal cortex [37,38]. Increases in grey matter volume within the parietal cortex also positively correlate with visual working memory performance [37]. Further, extensive AVG experience is also correlated with greater insular grey matter volume and functional connectivity between insular sub-regions [39]. There is also evidence of greater connectivity between frontal regions associated with cognition and visual areas in gamers, with an associated facilitation of visual texture discrimination [6].

To our knowledge, only one previous brain imaging study by Granek, Gorbet, and Sergio [40] has specifically examined brain activity associated with visuomotor processing in experienced AVG players. In particular, this study examined the effects of AVG experience in men on functional activity underlying several different visuomotor mappings. Most of our daily interactions with objects around us rely on a “standard” visuomotor mapping consisting of a saccade and an arm movement to the same location in space. For example, looking at and then reaching out to grasp a glass of water. Evidence suggests that in order to simplify the neurological processes involved in visual-to-motor transformations, the spatial locations of our gaze and reach are coupled by default [41–45]. However, computer and console-based video games rely upon non-standard visuomotor transformations where the targets of eye movements and arm movements are spatially decoupled. In other words, players of these games receive visual input from a vertical screen and then make spatially dissociated arm movements using the game controller to produce an outcome in the game. Therefore, it is likely that individuals who play a great deal of AVGs perform non-standard visuomotor transformations more often than their non-gamer peers. Indeed, using whole-brain comparisons of fMRI signal amplitude, Granek and colleagues observed that video game players had relatively less movement planning-related activity in regions of the posterior parietal cortex, and significantly greater activity in prefrontal regions during tasks requiring a “non-standard” visuomotor mapping, suggesting that high levels of AVG play evokes changes in the frontal-parietal circuitry underlying visually-guided action production.

A large proportion of the action video game training studies have done a good job of balancing the number of male and female study participants. These studies typically demonstrate benefits of AVG training in both sexes [14,17,46]. However, one sex often benefits from training more than the other, or complex interactions between sex and AVG exposure arise [15,24,25,47–50]. These results suggest that greater knowledge of potential sex-related differences in the effects of AVG exposure would be beneficial to our overall understanding of how these games change the human brain. In contrast to training studies, participants in studies examining the effects of extensive previous AVG exposure have been almost exclusively male. Globally, in nations where video game playing is popular, approximately half of all consumers of video games are female [51]. However, estimates are that females only make up approximately a quarter of the players of the action video game genre, and instead appear to be more likely to play puzzle and strategy-based video games [48,52]. Therefore, women with a long history of AVG experience may be in relatively shorter-supply than male gamers. Nevertheless, AVGs are the video game genre thought to convey the greatest improvement to the widest range of visuomotor- and attention-related skills.

The ultimate goal of a large proportion of AVG-related research is to develop tools for training and rehabilitative purposes to be used by both men and women. Many of the video game-like training and rehabilitative approaches currently being developed focus on improving visuomotor function. For example, there are training programs that either use video games directly or game-like interfaces to improve surgical skills in medical students [53,54], ones that aim to facilitate visuomotor rehabilitation after damage to the brain [55,56], and others that strive to attenuate age-related visuomotor decline [57–59]. Importantly, previous research demonstrates that there are sex-related differences in the functional neural correlates of visuomotor processing even when men and women are equally proficient at the motor tasks being examined [60–62]. Given what we know of the complex interactions between AVG exposure and the sex of an individual, and sex-related differences in functional brain activity associated with visuomotor processing, the inclusion of female participants in this body of research is imperative. Therefore, the purpose of the study presented here is to begin to fill two notable gaps in the AVG research literature. Namely, we use fMRI to expand the very limited knowledge of the effects of extensive action video game exposure on functional brain activity associated with visuomotor processing. Second, in order to include females in the literature on the effects of long-term exposure to AVGs, we perform this examination of visuomotor-related brain activity in women with an extensive history of AVG play.

## Methods

### Experiment 1—fMRI

#### Participants

Recruitment of participants was done using two sets of posters placed around the university campus. Both sets of posters stated a requirement for female participants in a study on eye-hand coordination. One set of posters aimed to recruit female AVG players. In order to limit the possibility of participants being influenced by the demand characteristics of the experiment [63], the posters did not specify what type of video games or how many hours of video game play were required for eligibility. This information was gathered from a questionnaire filled out by participants after recruitment but prior to acceptance into the study. Criteria to participate in the study as an action video game player required a history of AVG play for at least the previous 3 years with a self-reported approximate average of at least 10 hours per week of AVG play over the year prior to data collection. Games were considered AVGs if they required both quick processing of sensory information, and rapid responses. The most commonly played games reported by participants in the AVG group included first person shooters (Call of Duty, Borderlands, Left 4 Dead), racing games (Mario Kart, Grand Theft Auto, Need for Speed), fighting games (Super Smash Bros., Mortal Kombat), and action-adventure games (Tomb Raider, Red Dead Redemption, Assassin’s Creed). All participants listed several games belonging to more than one of these genres. The mean amount of AVG play reported was 17.1 +/- 9.2 SD hours per week. A second set of posters did not include any reference to video games at all and was used to gather non-AVG playing control participants for the study (also established through responses on questionnaires filled out after recruitment). Non-AVG players included in the study played less that 1 hour per week of AVGs for at least 3 years prior to data acquisition. The mean amount of AVG play reported by non-players was 0.2 +/- 0.4 SD hours per week. Several of these individuals did however play non-action video games (i.e. games that do not impose strict response time constraints or processing of stimuli presented for very brief durations) such as scrabble, solitaire and Sudoku. Questionnaires collected information on age, handedness, neurological medical history, and video game playing history.

In total, 20 participants were included in the experiment, 10 action video game players, and 10 individuals who did not play AVGs. The mean age of participants classified as gamers was 26.5 +/- standard deviation of 7.74 and the mean age of participants classified as non-gamers was 23.9 +/- 4.86 SD. The result of an independent, two-tailed t-test showed no significant differences in the mean ages of the two experimental groups (*t*_*18*_ = 0.90, *p* = 0.3801). The handedness of each participant was also quantified using the Oldfield Edinburgh Inventory [64]. All participants were right-hand dominant. The mean inventory score of the gamer group was 87.8 +/- 16.2 SD. The mean inventory score of the control group was 92.4 +/- 8.6. A two-tailed independent *t*-test did not reveal any statistically significant differences in handedness between the groups (*t*_18_ = 0.797, *p* = 0.436). All participants in the study had normal or corrected-to-normal vision and no history of neurological problems. Participants were reimbursed for their time at a rate of $15 per hour. The York University Research Ethics Board human participants subcommittee approved the protocol used in the experiment. The experimental protocol was also in compliance with the Declaration of Helsinki. All participants provided informed written consent prior to data collection.

#### Apparatus and stimuli

MRI data were acquired with a 3T Siemens Magnetom Avanto MRI system located at York University. In the scanner, participants lay supine with their heads tilted forward approximately 30 degrees using an angled plastic wedge placed under the head coil to allow participants to directly view stimuli. Stimuli were back-projected at a resolution of 1024 x 768 and a refresh rate of 60 Hz onto a plastic screen with a transparent touchpad mounted to it (Keytec Inc., Garland, Texas, USA; 17.0 cm x 12.8 cm). The screen was attached to the MRI bed at a distance where participants could comfortably make sliding hand movements on the screen between targets projected onto the screen with small movements of the wrist and lower arm. Tilting participants’ heads required a head coil arrangement such that the bottom half of a 12-channel receive-only head coil was used at the back of the head (integrated into the head cradle) and a 4-channel flex coil was placed over the forehead to collect signal from the anterior part of the brain [42,65]. Participants’ heads were secured into place using foam padding. Participants’ right upper arms were also secured into place with a velcro strap around the upper body to minimize the translation of task-related arm movement to the head.

T1-weighted anatomical images were collected using 192 slices in the sagittal orientation, TR = 1900 ms, TE = 2.44 ms, flip angle = 9 degrees, FoV = 240 mm, and a voxel dimension of 0.9 x 0.9 x 1.0 mm. Functional T2*-weighted images were collected using gradient echoplanar imaging with TR = 2500 ms, TE = 30 ms, flip angle = 90 degrees, FoV = 192 mm. Thirty nine slices were collected with a thickness of 3.0 mm and a voxel dimension of 2.0 x 2.0 x 3.0 mm using an interleaved acquisition sequence and zero gap between slices. This imaging protocol allowed us to collect data from the entire cortex of all participants. However, in most participants, this protocol only allowed us to collect functional data from the superior half to two thirds of the cerebellum.

Stimuli were produced using custom written software in MatLab (The MathWorks, Inc., Natick, MA, USA). A small, cross-shaped cursor was displayed on the screen during times in the paradigm when participants were required to have their hand on the screen. The touchpad was calibrated to ensure that the cursor remained below the participant’s fingertip and was therefore not visible to the participant. The cursor allowed us to visually validate the touchpad calibration and view the trajectory of hand movements made on the screen from a monitor in the MRI control room. Movements of the right eye were monitored using an MRI-compatible eye tracker (Avotec Eye Tracking Sytem RE-5721, Stuart, FL, USA; sampling rate of 60 Hz, spatial resolution of 0.1 degree of eye movement). However, tilting participants’ heads so that they could directly view targets projected on the screen resulted in difficulty positioning the eye tracker camera to obtain optimal eye tracking without blocking the view of stimuli. As a result, eye position data were sometimes intermittent due to the eyelid blocking the camera’s view of part of the pupil in most participants. Most data were reliable enough to verify the direction of eye movements but not eye movement latencies or precise end points of movements. The lights in the MRI room were kept dim to allow optimal viewing of projected stimuli while still allowing participants to see their own hand moving on the touchpad screen.

#### Experimental runs

**Fig 1:**
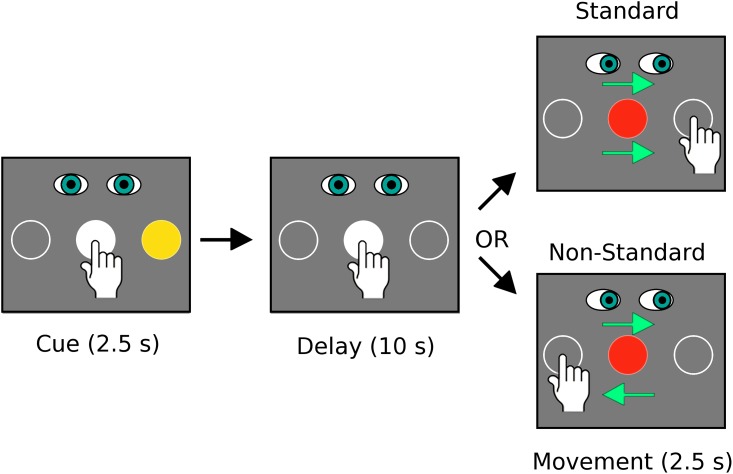
The standard and non-standard visuomotor mapping conditions. In both conditions participants were required to fixate on and touch the central target during the cue and delay periods. The central target turned from white to red to indicate the go signal for movement initiation. At this signal participants saccaded to and slid their finger on the touch screen to the cued target location in the Standard condition. In the Non-Standard condition participants also saccaded to the cued target location but slid their hand to the opposite target. Note that the conditions were the same for both Experiment 1 (fMRI) and Experiment 2 (behavioural) but in the second experiment the delay period was decreased from 10 seconds to a variable delay of 1.5 +/- 0.5 seconds.

Each participant performed 4 experimental runs. Runs consisted of 2 blocks of 10 slow event-related trials (one block for each of the two conditions, see Fig 1 and below for details of the conditions). The two blocks were presented in random order. Stimuli presented to participants were synchronized with the MRI pulse sequence by output from the MRI control computer to the stimulus presentation program. Each run began with 15 s of baseline data collection where participants fixated on a central dot. Each block began with an instruction screen that was presented for 5 s and informed the participant which of the two experimental conditions they would be performing in the following block of 10 trials. The instruction screen was followed by another 15 s of central fixation to allow brain activity to return to baseline levels prior to beginning the visuomotor task. Five seconds prior to the start of each trial, participants received a pre-cue warning consisting of the small central fixation dot expanding in diameter into a larger, hollow circle. Upon receiving this signal, participants moved their arm from a resting position on their abdomen to the centrally positioned circle on the touch screen and continued fixating this position with their eyes. The cue period of each trial consisted of the centre target turning from a hollow white circle to a filled white circle with two circular targets appearing peripherally on the left and right 70 mm away from the central target (centre to centre). The diameter of each target was 23 mm. The distance of the central target on the screen from the participant’s eyes was approximately 500 mm (depending on the size of the participant and the exact head position in the bore). This distance created a visual angle of approximately 8 degrees from central to peripheral targets. During the cue period, at random, one of the two peripheral targets was coloured yellow, indicating that it was the target of interest for the trial (the other target was a hollow white circle). Participants were required to remain fixated on and touching the central target during this cue period (2.5 s). After the cue period, an instructed-delay period began with the yellow circle of the cued target became a hollow circle identical to the non-cued target (10 s). During the delay period, the participant was required to continue central fixation with their hand touching the central target. After the delay period, a movement period (2.5 s) was initiated with the central target turning from white to red. At this “go signal”, participants were required to move their eyes to the target that had been coloured yellow during the cue period. The required hand movement depended on which of the two experimental conditions was being tested in a particular block (Fig 1). During the “Standard” visuomotor mapping condition, the hand was required to slide along the touch screen to the target that was yellow during the cue period (i.e. the eyes and hand moved to the same target). During the “Non-Standard” visuomotor mapping condition, the hand was required to slide along the touch screen to the target opposite the one that was yellow during the cue period (i.e. the eyes and hand moved to different targets in 180 degree opposite directions). Feedback was provided with the cued target turning green when the hand reached the correct target location. Note that the cursor on the screen remained below the participant’s finger tip during both visuomotor mapping conditions. Each block contained 10 trials of each condition (5 leftward and 5 rightward, in random order). Therefore, over the 4 experimental runs, each participant completed 40 trials of each of the 2 conditions.

Participants received training on the experimental tasks prior to imaging. After watching the experimenter demonstrate the tasks, each participant performed two blocks of each condition with 15 trials per block. Participants were also provided with an illustrated diagram of the experimental tasks several days prior to scanning. To minimize task-related head movements in the MRI, participants were instructed to make small, smooth movements in the scanner rather than initiating movements quickly. Hand movements were made using mainly motion at the wrist or small movements at the elbow if necessary.

#### In-magnet behavioural data

Eye-tracker and touch screen data were analyzed using custom-written software in Matlab (The MathWorks Inc., Natick, MA, USA). Individual trials were included in the data analysis if the eyes and arm both moved in the correct direction(s) without direction reversals. Trials were discarded if either effector moved away from the central target location prior to the go signal. Reaction times (RT) and movement times (MT) were calculated for the hand based on central target exit time and peripheral target entry time. Trial exclusion criteria did not have specific RT or MT thresholds for the in-magnet data since participants were encouraged to move slowly and smoothly to avoid creating motion artifacts in the MRI data. However, if the arm movement was not complete prior to the end of the movement period, the trial was excluded from analysis.

#### MRI data preprocessing

Analysis of MRI data was performed using BrainVoyager QX (v2.8, Brain Innovation, Maastricht, The Netherlands). Head motion (translation and rotation) was monitored in real-time in the control room during data acquisition to verify that any head movement did not exceed 2 mm in any direction. Of the 20 participants, 1 AVG player had to repeat 2 experimental runs and one non-player had to repeat 1 experimental run due to excessive head motion.

Preprocessing of functional data included motion correction using a trilinear/sinc interpolation algorithm with the volume closest to the time of the anatomical scan (i.e. the first volume) used as the reference volume. Plots of head movement and movies of head movement over time were generated by the analysis software and visually inspected to confirm that all experimental runs included in the analysis were free from head movements over 2 mm and were free from obvious scanner-related artifacts. After application of the motion correction algorithm, slice time correction and linear trend removal were applied to each functional run. Spatial smoothing of 6 mm using a full width half maximum isotropic kernel was applied to each run. General linear model (GLM) design matrices were constructed from each participant’s stimulation protocol using a boxcar design convolved with a hemodynamic response function. Individual stimulation protocols were created for each experimental run. Within the stimulation protocols, separate predictors were defined for the cue periods, instructed-delay periods, and movement periods associated with each trial, as well as for the instruction periods that preceded each of the two blocks of trials in each run. Trials for the left and right targets were pooled together within each condition. Individual head motion profiles for each run (in 3 linear and 3 rotational directions) were added to associated stimulation protocols as predictors on non-interest to minimize motion artifacts.

#### Localizer run and isolation of regions of interest

Each participant performed a localizer run for the purpose of isolating regions of interest that were involved in production of visually-guided reaching movements. This localizer run was collected independently from the experimental runs that were used in subsequent data analyses and was therefore only used to isolate regions of interest. The localizer run contained 10 trials of the standard mapping condition and 10 trials of the non-standard mapping condition. As in the experimental runs, trials of each condition were grouped into 2 blocks and each block was preceded by a 5-second instruction screen to inform the participant which condition they would be performing in the upcoming block of trials.

Localizer run data were pre-processed with motion correction using a trilinear/sinc interpolation algorithm with the volume collected temporally closest to the acquisition of the T1-weighted anatomical MRI used as the reference volume. Head movement did not exceed 2mm in any direction for any of the participants in the study. Slice time correction and linear trend removal were also performed on each localizer run. The data were also spatially smoothed using a 6 mm full-width half-maximum isotropic kernel. Design matrices for general linear model (GLM) analysis were produced from each participant’s stimulation protocol using a boxcar design convolved with a hemodynamic response function. Within each participant’s stimulation protocol, separate predictors were defined for the cue periods, instructed-delay periods, and movement periods of each trial for both of the two conditions; however, trials for the standard and non-standard mapping conditions were pooled together to reveal areas active in both conditions. Predictors were also defined for each of the instruction periods preceding each of the two blocks of trials. Within each of the two conditions, trials toward the left and right targets were pooled together in stimulation protocols. The head motion profiles (3 linear and 3 rotational directions) were added to each participant’s stimulation protocol as predictors of non-interest.

Localizer run data were normalized to Talairach space. Runs collected for the AVG player group and the AVG non-player group were analyzed using a conjunction analysis to localize regions of interest that were active in both groups. Thresholding of the resulting statistical map used a false discovery rate approach (FDR) with *q* set to 0.05. The resulting regions of interest (ROIs) are summarized below in Table 1.

**Table 1:** Regions of interest localized across all participants.

Region of interest (Brodmann area designation of centre of gravity)	No. voxels	Mean Talairach coordinates of centre of gravity (*x*, *y*, *z*)
Left lateral premotor (BA 6)	3396	-38, -7, 56
Right lateral premotor (BA 6)	2328	29, -3, 55
Left medial premotor (BA 6)	1290	-7, 1, 57
Right medial premotor (BA 6)	303	7, 9, 57
Left & right medial premotor (BA 6)	3300	3, -14, 50
Left primary sensorimotor (BA 1)	7994	-34, -30, 57
Right primary sensorimotor (BA 1)	7514	29, -29, 61
Left parietal lobule (BA 39)	2896	-42, -71, 29
Right medial parietal (BA 7)	1238	2, -70, 36
Left middle occipital gyrus (BA 18)	10274	-25, -86, 0
Right middle occipital gyrus (BA 18)	8921	25, -86, -7
Left cuneus (BA 18)	6490	-7, -75, 8
Right cuneus (BA 18)	6763	7, -75, 8
Left posterior lobe of cerebellum	1020	-33, -73, -35
Right posterior lobe of cerebellum	771	17, -79, -32

#### Comparison of head motion between groups during experimental imaging runs

Since part of the analysis procedure involved comparing fMRI signals occurring after the go signal for initiating movement, we quantified in-scan head motion for comparison between the experimental groups. Head motion was calculated using Brainvoyager QX in x, y, and z directions of linear translation and in x, y, and z (pitch, roll, and yaw) directions of rotation for each volume over the course of each imaging run. Composite translation and composite rotation values over time were calculated separately by squaring each x, y, and z value, then summing the resulting squared values and then taking the square root of this sum at each time point. These measurements were combined across imaging runs for each participant. For each participant’s resulting composite translation and rotation values over time, the area under the curve was estimated using Riemann sums and used to test for differences in amounts of translational and/or rotational head motion between the AVG-player and non-player groups using two-tailed independent t-tests.

#### fMRI data analyses

Regions of interest (ROIs) derived from the mean Talairach normalized localizer runs of all participants (see Table 1) were applied to the experimental runs collected from participants. Experimental runs were left in native subject space but ACPC aligned. For each participant, base-line normalized beta weights were calculated for each model predictor using a random effects, general linear model (GLM) approach. Two-factor, mixed-effect ANOVAs with repeated measures were run with beta weights associated with the instructed-delay period of the standard and non-standard mapping conditions as the within subjects factor and experimental group (i.e. AVG players or non-players) as the between subjects factor.

Two post-hoc analyses were run on data from ROIs in which a significant between group difference was detected in the ANOVA analyses. For the first analysis, a linear regression was performed for each participant’s mean BOLD signal over the instructed-delay period versus self-reported estimates of average amounts of time spent playing action video games per week in the year prior to scanning. For the second post-hoc examination, event-related averaging of BOLD signal time-courses in these regions of interest was performed for each individual participant for the standard and non-standard visuomotor mappings such that epochs were time-locked to the onset of the instructed-delay period and extracted from 2 volumes prior to delay onset to 11 volumes after delay onset. Therefore, these averages included the cue period, instructed delay period, movement period and inter-trial intervals. All 4 experimental runs were pooled together for each participant to obtain event-related averages. For each participant, the peak beta weight after the Go signal was obtained from the event-related average as well as the volume number (relative to the onset of the delay period) at which this peak occurred. Peak fMRI beta weights after the Go signal were compared using two-factor, mixed-effect ANOVAS with repeated measures (i.e. experimental group as the between subjects factor, and visuomotor mapping as the within subjects factor). Similarly, the timing at which this peak occurred (i.e. the volume number after the onset of the instructed-delay period) was also compared using two-factor, mixed-effect ANOVAs with repeated measures. In addition, linear regression analyses were performed for the timing (i.e. volume number) at which the peak fMRI beta weight occurred after the Go signal versus self-reported estimates of average amounts of time spent playing action video games per week in the year prior to scanning were run.

## Results

### Experiment 1—fMRI

#### In-magnet behavioural data

The total number of reaching errors made in the magnet did not significantly differ between the AVG player and non-player groups (*F*_1, 18_ = 4.90, *p* = 0.25). There were also no significant differences in total number of errors between the two visuomotor mapping conditions (*F*_1, 18_ = 0.084, *p* = 0.78). The mean number of reaching errors across all 4 experimental imaging runs and both conditions that were made by AVG players was 1.4 errors +/- 1.8 SD and in the non-players 0.7 errors +/- 1.2 SD. Trials containing these errors were excluded from further analysis. Of this small number of excluded trials, there were four different types of errors: failing to complete the movement before the end of the trial (69.23% of the total amount of errors in AVG players and 62.96% of the total amount of errors in non-players), moving prior to the go signal (15.38% of the total amount of errors in AVG players and 22.22% of the total amount of errors in non-players), failing to initiate a movement, (0% of the total amount of errors in AVG players and 11.11% of the total amount of errors in non-players), and moving the arm in the wrong direction or making a direction reversal (15.38% of the total amount of errors in AVG players and 3.70% of the total amount of errors in non-players).

In the MRI, participants were instructed to make slow, smooth arm movements in order to minimize the translation of arm movements to the head. The mean reaction times (RT) for the two groups of participants were not significantly different (*F*_1, 18_ = 2.19, *p* = 0.16). There were also no significant differences in RT between the two visuomotor mapping conditions (*F*_1, 18_ = 1.8, *p* = 0.19). Mean RT values (pooled across visuomotor mapping conditions) were 1.15 s +/- 0.16 SD for the AVG players and 1.3 s +/- 0.28 SD for the non-players. Similarly, the mean movement times (MT) for the two groups of participants were not significantly different (*F*_1, 18_ = 0.29, *p* = 0.60) and there were also no significant differences in MT between the two visuomotor conditions (*F*_1, 18_ = 1.85, *p* = 0.19). Mean MT values (pooled across visuomotor mapping conditions) were 0.424 s +/- 0.215 SD for the AVG players and 0.374 s +/- 0.189 SD for the non-players.

As noted above, participant’s heads were tilted forward in the scanner to allow direct viewing of targets projected onto the screen. This head position made it difficult to position the eye tracker optimally. Eye position data were sometimes intermittent due to the eyelid blocking the camera’s view of part of the pupil in the majority of participants. Therefore, eye data were reliable enough to obtain the direction of movements but not eye movement latency or precise movement end points. In total, the percentage of trials in which the eye data did not contain enough information to infer the direction of eye movements was 13.5% in the AVG players and 19.5% in the non-players. The entire eye movement data set for one AVG player and one non-player were excluded from analysis. Of the remaining data, the number of direction reversals or movements to the wrong target did not significantly differ between the AVG players and non-players (*F*_1, 16_ = 0.90, *p* = 0.36) and there were also no significant differences in these measures between the two experimental conditions (*F*_1, 16_ = 0.36, *p* = 0.55). The mean number of eye movement errors (pooled across visuomotor conditions and all 4 experimental runs) were 1.67 +/- 2.25 for the AVG players and 0.95 +/- 1.42 for the non-players.

#### Comparison of head motion between groups

**Fig 2:**
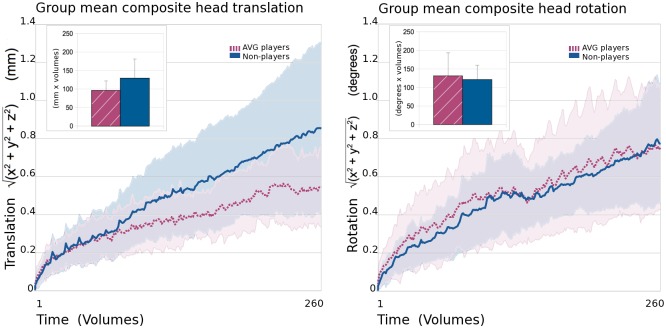
Translational and rotational head movement. Group mean composite (x, y, and z directions) translational head motion (left panel) and rotational head motion (right panel). Data from the action video game (AVG) player group are represented by dashed burgundy lines and non-player data are represented by solid blue lines. Standard deviations are shown in paler colours around main plotted lines. Comparisons of estimates of area under the curve (using Riemann sums) for the gamer (hatched burgundy) and non-gamer (blue) groups are shown inset at the top left of each panel. Error bars represent standard deviation. No significant group differences in area under the curve were detected indicating that the overall amount of head motion in the scanner did not significantly differ between the AVG player and non-player groups.

Two-tailed independent t-test comparisons of area under the curve (AUC) estimates for group mean head motion (composite translational motion and composite rotational motion) did not reveal significant differences between the gamer and non-gamer groups of participants (Fig 2). Mean AUC for translational head motion over experimental imaging run collection was 96 (mm x volumes) +/- 125.9 SD for the gamer group and 129 +/- 52.1 for the non-gamer group, t = 1.79, p = 0.09. Mean AUC for rotation head motion was 131 (degrees x volumes) +/- 62.4 for the gamer group and 121 +/- 38.8 for the non-gamer group, t = 0.43, p = 0.67.

#### Region of interest GLM comparisons

**Fig 3:**
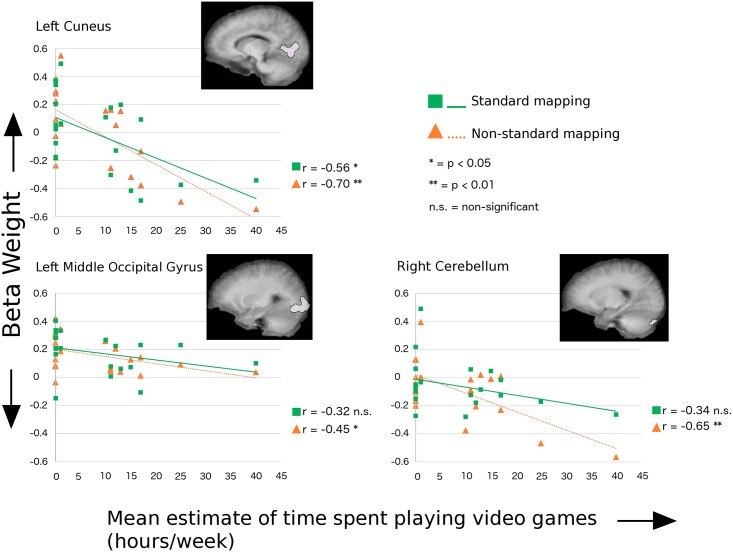
Relationship between preparatory BOLD signal and time spent playing. Linear regression results for mean instructed-delay epoch BOLD signal beta weights versus estimates of time spent playing action video games in regions of interest with significantly lower amplitude BOLD signal in the AVG player group compared with the non-player group. Regions of interest are displayed on each regression plot as overlays on the mean T1-weighted anatomical images for all participants. The Standard visuomotor mapping is represented by green squares and solid green lines. The Non-Standard visuomotor mapping is represented by orange triangles and dashed orange lines. See Table 2 for specific *p*-values.

Statistically significant differences in fMRI BOLD signal amplitudes associated with the instructed-delay period of each trial were observed between the gamer and non-gamer groups. The non-gamer group had significantly greater mean activity than the gamer group in regions of interest that included the left cuneus (*F*_1, 18_ = 6.61, *p* = 0.019), left middle occipital gyrus (marginally significantly different; *F*_1, 18_ = 4.35, *p* = 0.052), and the superior portion of the right posterior lobe of the cerebellum (*F*_1, 18_ = 6.96, *p* = 0.017). Linear regression analyses of mean fMRI beta weights during the instructed delay period versus self-reported estimates of average amounts of time spent playing action video games per week were run on ROIs in which a significant between group difference was observed in the ANOVA analyses. A significant negative correlation between estimates of time spent playing AVGs per week and mean beta weight during the delay period was observed in all tested regions for the non-standard visuomotor condition but only in the left cuneus for the standard visuomotor condition (see Fig 3 and Table 2).

**Table 2:** Linear regression of mean beta weights during the instructed-delay period versus estimated time spent playing video games each week.

Region of Interest	Standard mapping		Non-standard mapping	
	Pearson correlation coefficient (*R*)	*p*-value	Pearson correlation coefficient (*R*)	*p*-value
Left cuneus	-0.56	0.011	-0.70	0.00065
Left middle occipital gyrus	-0.32^a^	0.17	-0.45	0.053
Right posterior superior cerebellum	-0.34	0.14	-0.65	0.0012

^a^ Non-statistically significant results are shown with a grey background.

**Fig 4:**
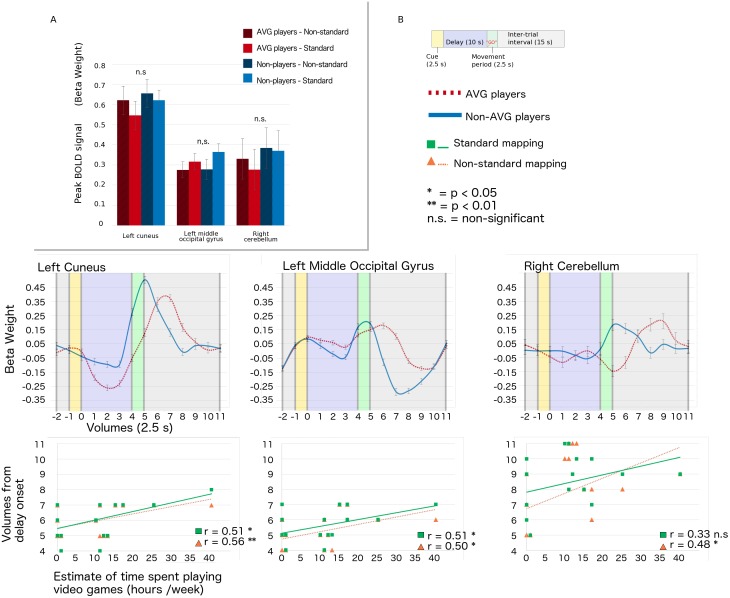
Relationship between the timing of peak BOLD response and time spent playing. A) Histogram of peak beta weights of trials of the standard and non-standard visuomotor mapping tasks for action video game (AVG) players (burgundy) and non-players (blue) for the full trial time course. Only regions of interest in which AVG players had significantly lower amplitude BOLD signal than non-players in the instructed-delay epoch of trials are shown. Error bars represent standard deviation. No statistically significant differences in peak signal amplitude were observed between experimental groups or visuomotor tasks. B) Top plots: Event-related averages of fMRI activity over trials time-locked to the onset of the delay period. Time-courses associated with the standard and non-standard mapping tasks have been pooled for visual clarity. Error bars represent standard deviation. Time-courses for AVG players shown with burgundy dashed lines and non-players shown with blue solid lines. Bottom plots: Linear regression results showing relationships between the timing of peak BOLD signal and estimates of time spent playing AVGs. Results for the standard mapping task shown with green squares and solid lines. Results for the non-standard mapping task shown with orange triangles and dashed lines. See Table 3 for specific *p*-values.

For the regions of interest described above, in which comparisons revealed that the gamer group had significantly lower amplitude BOLD signal than the non-gamer group over the instructed-delay period, peak beta weights associated with trials in the standard and non-standard conditions were obtained from event-related averages (time-locked to the onset of the delay period). Two-factor, mixed-model ANOVAs with visuomotor mapping condition as the within subjects factor and experimental group (i.e. AVG player or non-player) as the between subjects factor were run for mean peak beta weights for each of the ROIs. No significant main effects of mapping condition or group were observed (i.e. all *p*-values > 0.05, see Fig 4). Similarly, no significant interactions of mapping condition and visuomotor mapping were observed. These results indicate that despite lower BOLD signal amplitude in gamers during the delay period, the peak amplitude of fMRI signals (i.e. after the go signal, from event-related averages) were not significantly influenced by either the visuomotor mapping performed or self-reported AVG experience in any of the tested regions. That is, the neural signals ultimately reached the same amplitude over the course of carrying out the behaviour regardless of participant group or condition.

To reconcile the finding that the delay period signal amplitude was lower with AVG experience (something observed in previous MRI research on elite performers), yet overall the peak amplitude did not vary with experience, we examined the timing of the peak BOLD signal. The timing (in volumes from the onset of the delay period) at which peak beta weights occurred was also obtained from event-related averages of the BOLD signal in these regions. Linear regression analyses revealed significant relationships between the timing of peak activity in the standard mapping condition and estimates of time spent playing AVGs in the left cuneus, and the left middle occipital gyrus but not in the superior portion of the right posterior lobe of the cerebellum, see Table 3 and Fig 4). In particular, significant positive correlations were observed indicating that the peak of activity occurred later in individuals who spent more time playing AVGs. Significant relationships were also observed for the non-standard mapping condition in all regions tested. Thus when one examines the full time course of neural activity from initial instruction through action completion, one finds that the experience-related differences are a function of neural activity timing rather than overall neural activity amplitude in the relevant brain regions.

**Table 3:** Linear regression of the timing of mean peak beta weights versus estimated time spent playing action video games each week.

Region of Interest	Standard mapping		Non-standard mapping	
	Pearson correlation coefficient (*R*)	*p*-value	Pearson correlation coefficient (*R*)	*p*-value
Left cuneus	0.51	0.022	0.56	0.010
Left middle occipital gyrus	0.51	0.021	0.50	0.025
Right posterior lobe cerebellum	0.33^a^	0.16	0.48	0.032

^a^ Non-statistically significant results are shown with a grey background.

## Methods

### Experiment 2—Arm movement kinematics

In order to limit head motion artifacts in the fMRI data, participants of Experiment 1 were instructed to move their hands slowly and smoothly in the scanner. Therefore, to examine potential kinematic differences without these constraints, arm movements made by women who play action video games and those who do not were compared in a separate experiment as follows.

#### Participants

Recruitment of participants and inclusion criteria were identical to those described in Experiment 1. A total of 20 right-handed female participants were included in Experiment 2: 10 action video game players and 10 non-players of AVGs. Four participants in each experimental group also participated in Experiment 1. Questionnaires collected information on age, handedness, neurological medical history, and video game playing history. The mean amount of AVG play reported by gamer participants was 15.7 +/- 2.2 SD hours per week. Game preferences reported by this group of participants were very similar to that described by the participants in the MRI experiment. The mean amount of AVG play reported by non-gamer participants was 0.4 +/- 0.22 SD hours per week. As in Experiment 1, several of these individuals did play non-action video games such as scrabble and Sudoku. The mean age of participants classified as gamers was 26.0 +/- 7.2 SD and the mean age of participants classified as non-gamers was 24.9 +/- 6.9. The result of an independent, two-tailed t-test showed no significant differences in the mean ages of the two experimental groups (*t*_*18*_ = 0.350, *p* = 0.7304). All participants were right hand dominant and had normal or corrected-to-normal vision and no history of neurological problems. Participants were reimbursed for their time at a rate of $15 per hour.

#### Apparatus and stimuli

Participants were seated in front of a computer monitor in a quiet, dimly lit room. A transparent touch screen with a sampling rate of 100 Hz (Keytec Inc., Garland, Texas, USA) was installed over the screen of the monitor to record the trajectories of hand movements. Participants wore earplugs during data collection and a glove on their right hand to facilitate the production of smooth movements on the touch screen. Except for the timing of stimulus presentation, the experimental conditions tested were identical to the “Standard” and “Non-Standard” visuomotor mapping conditions performed in the MRI, and shown in Fig 1. Additionally, unlike the instructions received by participants of Experiment 1, participants in Experiment 2 were instructed to move to target locations as quickly and accurately as possible after receiving the go signal for movement initiation in each trial. Trials of the visuomotor mapping conditions in this experiment consisted of a 2.5 s cue period, followed by a 1.5 +/- 0.5 s instructed-delay period, and then by a 2.5 s movement period. Inter-trial intervals were 2.0 s in length. As in the fMRI portion of the study, trials of each condition were grouped into 2 blocks, each preceded by an instruction screen that remained visible for 5.0 s. Each block contained 20 trials of one of the two conditions; therefore each experimental run contained 40 trials in total. Within each run, the two conditions were presented in random order. Training of each participant consisted of the performance of one experimental run (i.e. 20 trials of each condition). After training, each participant performed 4 experimental runs, giving a total of 80 trials of each of the two conditions. The size and distance of targets were identical to those in the fMRI experiment (i.e. the diameter of each target was 23 mm with a distance of 70 mm between the centres of the central and peripheral targets and an approximate distance of 500m from the participant’s eyes and the central target on the screen).

#### Behavioural data analysis

Behavioural data analysis was done using custom written software (Matlab, The Mathworks, Natick, MA, USA). Trials were included in data analysis if the reaction time was between 120 and 1000 ms and the movement time did not exceed the duration of the trial movement period. In addition, in successful trials, participants moved in the correct direction without direction reversals between targets and movements did not occur prior to the go signal. Reaction times (RT) were defined as the time after the appearance of the go signal at which the velocity of the hand movement on the touch screen first reached 10% of the peak velocity obtained for the trial. Ballistic movement times (MT) were calculated as the amount of time between the RT and the time at which the velocity of the movement first decreased back down to 10% of the peak velocity of the trial. Corrected MT was calculated as the amount of time between the RT and the conclusion of any small correctional movements of the arm made after the first ballistic movement. Ballistic and corrected absolute errors (AE, movement accuracy) were calculated as the absolute distance between the average movement endpoint and the actual central location of the target. Variable errors (VE, movement precision) were calculated as the distance between the endpoints of movements from individual trials and the mean of these endpoint locations. Two-factor, mixed-effects, repeated-measures ANOVAs were performed for each variable with Group (i.e. gamer or non-gamer) as a between-subjects factor and visuomotor mapping (i.e. standard or non-standard) as a fixed-effect, within-subjects factor.

## Results

### Experiment 2—Behavioural data

**Fig 5:**
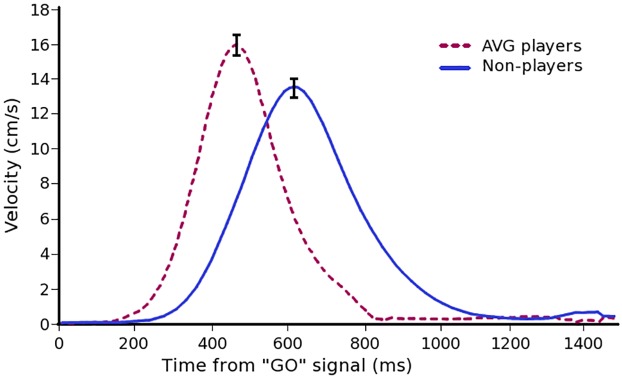
Kinematic measures of arm movements in AVG players and non-players. Mean arm movement velocity profiles after the “Go” signal for the AVG players (dashed burgundy line) and non-players (solid blue line). Velocity profiles are pooled for the Standard and Non-standard visuomotor conditions. Error bars represent SEM.

To avoid head motion artifacts, arm movements made within the MRI scanner were made slowly and were produced using mainly movement at the wrist joint. Therefore, in a second experiment, we examined movement kinematics in AVG players compared with non-players without MRI-imposed limitations. The results of this second experiment demonstrated that AVG players had significantly faster reaction times, peak movement velocities, ballistic movement times (MT) and corrected MT relative to non-players (see Table 4 for specific values and Fig 5 for group mean velocity profiles). Corrected MT was significantly longer in the non-standard condition relative to the standard condition in both groups of participants. In addition, the maximum velocity of movements was slightly significantly faster for the standard condition relative to the non-standard condition in both groups. End-point errors were measured relative to the centre of the target. Ballistic and corrected absolute errors (AE) were significantly larger in the non-standard condition relative to the standard condition; however, there were no significant differences in AE (either ballistic or corrected) between the AVG player and non-player groups. Ballistic and corrected variable errors (VE) were significantly larger in the non-standard condition relative to the standard condition. In addition, the AVG player group had significantly larger VE (ballistic and corrected) than the non-player group; however, mean values still fell with in the boundaries of target diameters.

**Table 4:** Mean values of kinematic measures collected in Experiment 2 in the AVG player and non-player groups for the standard and non-standard visuomotor mapping conditions.

	AVG players		Non-players		*F* value (*df* = 1,18) a) Main effect group b) Main effect condition c) Interaction (group x condition)	*P* value
	Standard	Non-Standard	Standard	Non-Standard		
Reaction time (ms)	204.2 +/- ^a^ 58.5	207.7 +/- 41.5	329.8 +/- 51.1	324.0 +/- 71.8	a) 24.60 b) 0.029 c) 0.420	0.0001 * 0.87 0.52
Maximum velocity (cm/s)	16.1 +/- 1.9	15.9 +/- 1.7	13.7 +/- 1.7	13.4 +/- 1.7	a) 10.15 b) 4.31 c) 0.61	0.0051 * 0.053* 0.44
Ballistic movement time (ms)	590.1 +/- 83.7	596.1 +/- 82.1	711.0 +/- 126.3	723.1 +/- 130.1	a) 6.65 b) 3.53 c) 0.41	0.019 * 0.077 0.53
Corrected movement time (ms)	672.3 +/- 111.8	716.9 +/- 136.2	831.9 +/- 123.0	890.9 +/- 142.1	a) 8.98 b) 12.24 c) 0.24	0.0077 * 0.0026 * 0.63
Ballistic absolute error (mm)	5.8 +/- 1.4	6.6 +/- 2.2	5.3 +/- 1.4	6.1 +/- 1.9	a) 0.46 b) 8.33 c) 0.008	0.51 0.0098 * 0.93
Corrected absolute error (mm)	5.3 +/- 1.4	5.9 +/- 1.8	5.0 +/- 1.4	5.4 +/- 1.3	a) 0.46 b) 4.74 c) 0.35	0.51 0.043 * 0.56
Ballistic variable error (mm)	6.4 +/- 1.5	8.2 +/- 3.0	4.9 +/- 1.2	5.6 +/- 1.9	a) 7.14 b) 7.10 c) 1.42	0.016 * 0.016 * 0.25
Corrected variable error (mm)	5.8 +/- 1.6	7.5 +/- 2.4	4.7 +/- 1.3	5.0 +/- 1.7	a) 6.30 b) 6.42 c) 3.07	0.022 * 0.021 * 0.097

^a^ All measures are reported +/- standard deviation.

* Statistically significant

## Discussion

The study presented here looks at the neural correlates of visually-guided arm movement in women with an extensive history of playing action video games. To our knowledge, this is the first examination of brain activity in highly experienced, female AVG players and only the second direct examination of visuomotor-related brain activity in gamers in general. Our main findings were as follows. We observed less preparatory-related activity (i.e. prior to movement execution) in female AVG players relative to non-AVG players in regions of the occipital cortex and cerebellum. Further, during this movement preparation period, there was a significant negative relationship between self-reported hours spent playing and the amplitude of the BOLD signal. However, the results suggest that these group differences in signal amplitude may actually reflect differences in the *time-course* of task-related BOLD activity, rather than differences solely in signal amplitude across the full course of behaviour. Namely, more time spent playing AVGs was associated with later peak BOLD activity in spite of the fact that women who played AVGs had significantly faster visually-guided responses than women who did not. We discuss these findings in detail in the sections below.

### The relationship between visuomotor brain activity and time spent playing action video games

The experiments presented here used two visuomotor mapping conditions. In the standard mapping task the eyes and hand moved to the same target location while in the non-standard task the eyes and hand moved to opposite target locations. Trials of each task contained an instructed-delay period after target presentation. Preparatory-related activity associated with this delay could be examined prior to the “go” signal for movement onset. The imaging data revealed that relative to women who do not play action video games, women who regularly play AVGs have significantly less preparatory activity in the left cuneus, the left middle occipital gyrus (MOG), and in the superior portion of the posterior lobe of the right cerebellar hemisphere. The amount of activity in these regions was negatively correlated to self-reported estimates of time spent playing AVGs. In other words, the greater the amount of time spent playing, the lower the mean amplitude of the BOLD signal during the delay period prior to motor execution. This relationship existed in all of these regions for both the standard and non-standard visuomotor mapping conditions, with the exception of the right cerebellum for which this relationship was not significant in the standard condition. Higher correlations were found for the non-standard condition in all cases (see Fig 3). Therefore, in agreement with our hypothesis, these data suggest that while AVG play has a significant effect on preparatory activity for visually-guided arm movement in general, these games may most impact brain activity associated with the preparation of non-standard tasks in which the targets of the eye and arm movements are spatially dissociated. This result makes sense given that computer and console-based games require non-standard mappings in which hand movements on the game interface (e.g. joystick, controller, keyboard) control non-spatially aligned visual targets that are typically viewed on a vertical screen. Therefore, experienced AVG players would be expected to have more exposure to non-standard visuomotor mappings than their non-AVG playing peers and consequently, different patterns of activity in associated neural circuitry. Importantly, the task used in the present study was not itself an action video game, but rather a task meant to study non-standard visuomotor performance more generally. That the experienced AVG players nevertheless demonstrated patterns of efficiency indicates that the neural mechanisms controlling their eye-hand coordination are used for motor behaviours outside of their trained skill set. Such a finding supports the generalizability of AVG training for visuomotor control training and rehabilitation.

The cuneus and cerebellar regions described here have previously been demonstrated to distinguish between standard and non-standard visuomotor mappings [42]; thus making them good candidate members of the visuomotor circuitry that is likely to be affected by AVG history. The large left cuneus ROI (medial BA 18) likely contained several sub-regions including V6A, V6, and V3A, and while these sub-regions all possess unique characteristics, they are also all highly interconnected [66,67] and are all responsive during eye and/ or arm movements [68–72]. Further, these regions are sensitive to visual motion and appear to use these signals to maintain perceptual stability [73] and to extract information about the spatial location of objects relative to the observer [71]. The cuneus is thought to form a hub from which parieto-prefrontal, parieto-premotor, and parieto-medial temporal pathways diverge [67,74]. Therefore this region possesses a number of characteristics that make it ideally situated for integration of the visual signals needed to coordinate the eye and arm during visually-guided movements in a dynamic environment [66,67]. Consistent with the findings reported here in the cuneus, others have reported that training on a joystick-controlled video game was associated with decreased activity in the cuneus [75].

The region of the cerebellum found to be active here is associated with coordinating the temporal relationship between eye and arm movements [76]. Further, this region likely has a role in the ability to successfully divide attention between spatially dissociated targets for eye and arm movements during the performance of non-standard visuomotor mappings [42,77]. In agreement with our observations of decreased preparatory activity in the posterior lobe of the cerebellum in experienced gamers, Balsters and Ramnani [78] also observed experience-related decreases in preparatory-related activity in this region during a non-standard visuomotor mapping between arbitrary symbols and finger movements.

In comparison with the cuneus and cerebellum, relatively less is known about the specific roles of the middle occipital gyrus in visuomotor control; however, this region is known to be active during visually guided arm movements [79,80]. In the context of the data reported here, it is interesting to note that higher grey matter volume in this region is associated with higher sensitivity to deviation of movement trajectories from a straight line during a visually-guided joystick movement task [81], establishing another connection between this region and a non-standard visuomotor task.

The finding of less activity in the left cuneus, left MOG, and right posterior lobe of the cerebellum in avid AVG players is consistent with other studies that have shown that expertise in a motor skill is associated with a reduction in corresponding brain activity [82–88]. Findings of less activity associated with a more highly skilled group are typically interpreted as representing greater neural efficiency. In other words, superior performance is achieved with less brain activity. However, upon examining the entire time-course of brain activity (i.e. rather than just activity associated with the delay epoch) in the regions with significantly lower preparatory BOLD signal in the gamers, it becomes apparent that decreased activity may not be the full story. In particular, the time-course of brain activity in the regions reported here appears to be shifted in the gamer group so that the peak of activity occurs later than it does in the non-gamer group (see Fig 4b). Indeed, significant relationships exist in these areas such that the more time an individual reports playing AVGs, the later the peak of activity occurs. The significant relationships between delay-related signal amplitude and the timing of peak signal amplitude with self-reported estimates of time spent playing AVGs were all very consistent in the regions tested; however, it is important to point out that a sample size of 20 participants is relatively low and these results should ideally be confirmed in a larger population of individuals. Comparisons of the amplitude of the mean peaks of BOLD activity between the two groups yields no significant differences. Taken together, these data suggest that the apparent decrease in preparatory activity during the instructed-delay epoch is likely due to the onset of increases in task-related BOLD signal occurring mainly after the completion of this epoch in the gamer group. This finding in turn suggests that the onset of task-related neural activity occurs closer to the beginning of movement execution in the AVG players than it does in their non-playing peers. We suggest that the currently-held notion of neural efficiency associated with elite performance is not solely a function of reduced neural activation, but a more complex pattern of neural activation and timing that allows final motor commands to be executed closer to the actual behavioural onset.

It should be noted that examining fMRI data collected during and after the arm movement of each trial increases the chance of task-related motion artifacts contributing to detected signals [89]. However, a great deal of effort was made to exclude task-related motion from contributing to the findings reported here. To help prevent arm movements from translating to the head, participants’ upper arms were immobilized with a strap and they were trained to make their arm movements in the scanner slowly and smoothly using only motion at the wrist if possible, or very slight motion at the elbow if necessary. No experimental runs in which head motion exceeded 2 mm in any direction were included in data analyses. Even so, head motion parameters associated with each run were included in stimulation protocols as predictors of no-interest. Further, head motion was compared between the AVG player group and the non-player group revealing no significant group-related differences in overall amounts of head motion. Therefore, group-related differences reported here are very unlikely to be a result of one group moving their heads in the scanner more than the other. A second caveat that must be kept in mind when considering the findings reported here is that compared to the speed of neural processing in the brain, the temporal resolution of the BOLD signal is quite poor. The data reported here were collected with a TR of 2.5 seconds and can therefore only provide an approximation of the timing of peak activity that does not approach the true rate of neural activity in the brain. Future examinations using a technique with a superior temporal resolution such as electroencephalography should be performed to confirm the results presented here. Additionally, it is important to again note that since this study only included 10 participants per group, these results should ideally be confirmed using a larger sample of participants.

The second experiment presented in this study had participants perform the same visuomotor mapping tasks that were performed in the scanner but without the limitation of having to produce slow movements that did not translate from the arm to the head. It should be noted that many of the participants in the second experiment differed from those in the first (4 participants from the AVG and 4 from the non-AVG player group participated in both studies). Participants were instructed to make movements as quickly and accurately as possible. The data demonstrated that women who played AVGs had significantly faster reaction times and maximum velocities of arm movements. Further, they completed the movements more quickly and did so as accurately as the non-gamer participants (i.e. absolute movement endpoint errors did not differ between the groups). The end point locations produced by the AVG group were significantly more variable (i.e. higher variable error values) than those produced by the non-gamers. However, because mean endpoints were located within the boundaries of the target for both groups of individuals, this difference may not be particularly meaningful. Rather, the experienced gamers may use a strategy of calibrating their movements to be as fast as possible while still maintaining functional target success. The observation of faster movements in the AVG player group is consistent with the observations of several other studies on AVG players [36,90,91]. Taken together with the imaging results, these findings suggest that the observed shift of the time course of brain activity seen in the AVG player group may represent a neural signature of superior motor performance in highly skilled individuals.

### The potential for sex-related differences in the effects of AVG play on the human brain

To our knowledge, this study is only the second direct examination of visuomotor-related brain activity in highly experienced action video game players. The first study (which included the authors of the current study) used fMRI to look at progressively more complex visuomotor mappings in male AVG players [40]. In this study by Granek and colleagues, the “rotated hand movement” visuomotor mapping was similar to the non-standard condition in the current study. After an instructed-delay period, participants moved their eyes to a cued target location and their hand 180 degrees in the opposite direction. However, the Granek study limited collection of MRI data to the frontal and parietal cortical regions and therefore did not examine the occipital or cerebellar regions in which the current study found significant differences between the female AVG player and non-player groups. Further, the study only examined motor preparatory-related activity in the instructed-delay portion of trials and did not examine whether or not relationships existed between self-reported amounts of time spent gaming and levels of activity. Therefore, the comparisons that can be made between that study and the one presented here are limited by these factors. However, consistent with the findings reported here, the Granek study found significantly lower preparatory-related activity in the AVG playing group in a left parietal-occipital sulcus region overlapping the superior portion of the left cuneus ROI reported here.

Overall, the findings of the Granek study of male AVG players and the study of female AVG players described here are consistent to the limited extent that they may be compared. However, these similarities do not preclude the necessity of including female participants in this field of research. Men and women have significantly different patterns of brain activity underlying the production of visually-guided arm movements even when both groups are equally proficient at the task [60,61]. These sex-related differences take the form of a more bilateral distribution of activity in women relative to men, along with the presence of regions that are more active in one group or the other. Further, the more bilateral distribution of brain activity in women may represent greater functional redundancy for motor planning relative to men [62]. This finding may in turn suggest a potential explanation for observations that the clinical signs of brain damage can differ depending on the sex of an individual [92–98]. Findings such as these are likely relevant and important for optimal implementation of video game-like training and rehabilitation paradigms. This is especially likely within the context of the many demonstrations of complicated interactions between AVG exposure and the sex of a participant [15,24,25,47–50]. Further, given that only approximately 25% of AVG players are women [48, 52], task designers will have to put careful thought into creating action video game-like systems that inspire compliance in both sexes.

It is clear from the findings reported here and in many other studies that AVGs have the potential to evoke neural plasticity resulting in a variety of skill enhancements that may be transferable to non-game-related activities. Including women in AVG research is essential to gain a full understanding of how these games change the human brain, and how we might use these potential changes in the context of skill enhancement during training and skill rehabilitation after brain injury. Ultimately, it will be important not just to include female participants in this field of research but also to directly compare results between the sexes to gain a full understanding of how AVGs affect the brain.

## References

[1] Bavelier D, Davidson RJ. Games to do you good. Nature. 2013;494: 8–9. doi: 10.1038/494425a 23446401 10.1038/494425a

[2] Dye MWG, Green CS, Bavelier D. The development of attention skills in action video game players. Neuropsychologia. 2009;47: 1780–1789. doi: 10.1016/j.neuropsychologia.2009.02.002 19428410 10.1016/j.neuropsychologia.2009.02.002 PMC2680769

[3] Hubert-wallander B, Green CS, Bavelier D. Stretching the limits of visual attention: the case of action video games. 2011; doi: 10.1002/wcs.116 26302012 10.1002/wcs.116

[4] Latham AJ, Patston LLM, Tippett LJ. The virtual brain: 30 years of video-game play and cognitive abilities. Front Psychol. 2013;4: 629 doi: 10.3389/fpsyg.2013.00629 24062712 10.3389/fpsyg.2013.00629 PMC3772618

[5] Gopher Daniel, Weil Maya, Bareket T. Transfer of Skill from a Computer Game Trainer to Flight. Hum Factors. 1994;36: 387–405.

[6] Kim Y-H, Kang D-W, Kim D, Kim H-J, Sasaki Y, Watanabe T. Real-Time Strategy Video Game Experience and Visual Perceptual Learning. J Neurosci. 2015;35: 10485–92. doi: 10.1523/JNEUROSCI.3340-14.2015 26203143 10.1523/JNEUROSCI.3340-14.2015 PMC6605119

[7] Latham AJ, Patston LLM, Tippett LJ. The precision of experienced action video-game players: Line bisection reveals reduced leftward response bias. Attention, Perception, Psychophys. 2014;76: 2193–2198. doi: 10.3758/s13414-014-0789-x 25341651 10.3758/s13414-014-0789-x

[8] Bejjanki VR, Zhang R, Li R, Pouget A, Green CS, Lu Z-L, et al Action video game play facilitates the development of better perceptual templates. Proc Natl Acad Sci. 2014;111: 16961–16966. doi: 10.1073/pnas.1417056111 25385590 10.1073/pnas.1417056111 PMC4250112

[9] Bavelier D, Green CS, Pouget A, Schrater P. Brain Plasticity Through the Life Span: Learning to Learn and Action Video Games. Annu Rev Neurosci. 2012;35: 391–416. doi: 10.1146/annurev-neuro-060909-152832 22715883 10.1146/annurev-neuro-060909-152832

[10] Boot WR, Champion M, Blakely DP, Wright T, Souders DJ, Charness N. Video games as a means to reduce age-related cognitive decline: attitudes, compliance, and effectiveness. Front Psychol. 2013;4: 31 doi: 10.3389/fpsyg.2013.00031 23378841 10.3389/fpsyg.2013.00031 PMC3561600

[11] Green CS, Pouget A, Bavelier D. Improved Probabilistic Inference as a General Learning Mechanism with Action Video Games. Curr Biol. Elsevier Ltd; 2010;20: 1573–1579. doi: 10.1016/j.cub.2010.07.040 20833324 10.1016/j.cub.2010.07.040 PMC2956114

[12] Green CS, Bavelier D. Action-video-game experience alters the spatial resolution of vision: Research article. Psychol Sci. 2007;18: 88–94. doi: 10.1111/j.1467-9280.2007.01853.x 17362383 10.1111/j.1467-9280.2007.01853.x PMC2896830

[13] Li R, Polat U, Makous W, Bavelier D. Enhancing the contrast sensitivity function through action video game training. Nat Neurosci. 2009;12: 549–551. doi: 10.1038/nn.2296 19330003 10.1038/nn.2296 PMC2921999

[14] Dorval M, Pépin M. Measure of Spatial Visualization. Percept Mot Ski. 1986;62: 159–162. 10.2466/pms.1986.62.1.159 3960656

[15] Feng J, Spence I, Pratt J. Playing an action video game reduces gender differences in spatial cognition. Psychol Sci. 2007;18: 850–5. doi: 10.1111/j.1467-9280.2007.01990.x 17894600 10.1111/j.1467-9280.2007.01990.x

[16] Boot WR, Kramer AF, Simons DJ, Fabiani M, Gratton G. The effects of video game playing on attention, memory, and executive control. Acta Psychol (Amst). Elsevier B.V.; 2008;129: 387–398. doi: 10.1016/j.actpsy.2008.09.005 18929349 10.1016/j.actpsy.2008.09.005

[17] Green CS, Bavelier D. Action video game modifies visual selective attention. Nature. 2003;423: 534–7. doi: 10.1038/nature01647 12774121 10.1038/nature01647

[18] Green CS, Bavelier D. Effect of action video games on the spatial distribution of visuospatial attention. J Exp Psychol Hum Percept Perform. 2006;32: 1465–1478. doi: 10.1037/0096-1523.32.6.1465 17154785 10.1037/0096-1523.32.6.1465 PMC2896828

[19] Karle JW, Watter S, Shedden JM. Task switching in video game players: Benefits of selective attention but not resistance to proactive interference. Acta Psychol (Amst). Elsevier B.V.; 2010;134: 70–78. doi: 10.1016/j.actpsy.2009.12.007 20064634 10.1016/j.actpsy.2009.12.007

[20] Irons JL, Remington RW, McLean JP. Not so fast: Rethinking the effects of action video games on attentional capacity. Aust J Psychol. 2011;63: 224–231. doi: 10.1111/j.1742-9536.2011.00001.x

[21] Murphy K, Spencer A. Playing video games does not make for better visual attention skills. J Artic Support Null Hypothesis. 2009;6: 1–20.

[22] Haier RJ, Siegel BVJ, MacLachlan A, Soderling E, Lottenberg S, Buchsbaum MS. Regional glucose metabolic changes after learning a complex visuospatial/motor task: a positron emission tomographic study. Brain Res. 1992;570: 134–143. doi: 10.1016/0006-8993(92)90573-R 1617405 10.1016/0006-8993(92)90573-r

[23] Lee H, Voss MW, Prakash RS, Boot WR, Vo LTK, Basak C, et al Videogame training strategy-induced change in brain function during a complex visuomotor task. Behav Brain Res. Elsevier B.V.; 2012;232: 348–357. doi: 10.1016/j.bbr.2012.03.043 22504276 10.1016/j.bbr.2012.03.043

[24] Hoeft F, Watson CL, Kesler SR, Bettinger KE, Reiss AL. Gender differences in the mesocorticolimbic system during computer game-play. J Psychiatr Res. 2008;42: 253–258. doi: 10.1016/j.jpsychires.2007.11.010 18194807 10.1016/j.jpsychires.2007.11.010

[25] Greenfield PM, Brannon C, Lohr D. Two-Dimensional Representation of Movement Throbgh Three- Dimensional Space : The Role of Video Game Expertise. J Appl Dev Psychol. 1994;15: 87–103.

[26] Buckley D, Codina C, Bhardwaj P, Pascalis O. Action video game players and deaf observers have larger Goldmann visual fields. Vision Res. Elsevier Ltd; 2010;50: 548–556. doi: 10.1016/j.visres.2009.11.018 19962395 10.1016/j.visres.2009.11.018

[27] Li R, Polat U, Scalzo F, Bavelier D. Reducing backward masking through action game training. J Vis. 2010;10: 1–13. doi: 10.1167/10.14.33 21191129 10.1167/10.14.33

[28] Sungur H, Boduroglu A. Action video game players form more detailed representation of objects. Acta Psychol (Amst). Elsevier B.V.; 2012;139: 327–334. doi: 10.1016/j.actpsy.2011.12.002 22266223 10.1016/j.actpsy.2011.12.002

[29] Greenfield PM, de Winstanley P, Kilpatrick H, Kaye D. Action video games and informal education: effects on strategies for dividing visual attention. J Appl Dev Psychol. 1994;15: 105–124.

[30] Green CS, Bavelier D. Enumeration versus multiple object tracking: the case of action video game players. Cognition. 2006;101: 217–245. doi: 10.1016/j.cognition.2005.10.004 16359652 10.1016/j.cognition.2005.10.004 PMC2896820

[31] Chisholm JD, Kingstone A. Action video game players’ visual search advantage extends to biologically relevant stimuli. Acta Psychol (Amst). Elsevier B.V.; 2015;159: 93–99. doi: 10.1016/j.actpsy.2015.06.001 26071923 10.1016/j.actpsy.2015.06.001

[32] Mishra J, Zinni M, Bavelier D, Hillyard SA. Neural basis of superior performance of action videogame players in an attention-demanding task. J Neurosci. 2011;31: 992–8. doi: 10.1523/JNEUROSCI.4834-10.2011 21248123 10.1523/JNEUROSCI.4834-10.2011 PMC6632922

[33] Bialystok E. Effect of bilingualism and computer video game experience on the Simon task. Can J Exp Psychol. 2006;60: 68–79. doi: 10.1037/cjep2006008 16615719 10.1037/cjep2006008

[34] Clark K, Fleck MS, Mitroff SR. Enhanced change detection performance reveals improved strategy use in avid action video game players. Acta Psychol (Amst). Elsevier B.V.; 2011;136: 67–72. doi: 10.1016/j.actpsy.2010.10.003 21062660 10.1016/j.actpsy.2010.10.003

[35] Donohue SE, Woldorff MG, Mitroff SR. Video game players show more precise multisensory temporal processing abilities. Atten Percept Psychophys. 2010;72: 1120–9. doi: 10.3758/APP.72.4.1120 20436205 10.3758/APP.72.4.1120 PMC3314265

[36] Bavelier D, Achtman RL, Mani M, Focker J. Neural bases of selective attention in action video game players. Vision Res. Elsevier Ltd; 2012;61: 132–143. doi: 10.1016/j.visres.2011.08.007 21864560 10.1016/j.visres.2011.08.007 PMC3260403

[37] Tanaka S, Ikeda H, Kasahara K, Kato R, Tsubomi H, Sugawara SK, et al Larger Right Posterior Parietal Volume in Action Video Game Experts: A Behavioral and Voxel-Based Morphometry (VBM) Study. PLoS One. 2013;8: 4–9. doi: 10.1371/journal.pone.0066998 23776706 10.1371/journal.pone.0066998 PMC3679077

[38] Kühn S, Gallinat J. Amount of lifetime video gaming is positively associated with entorhinal, hippocampal and occipital volume. Mol Psychiatry. 2014;19: 842–7. doi: 10.1038/mp.2013.100 23958958 10.1038/mp.2013.100

[39] Gong D, He H, Liu D, Ma W, Dong L, Luo C, et al Enhanced functional connectivity and increased gray matter volume of insula related to action video game playing. Sci Rep. 2015;5: 9763 doi: 10.1038/srep09763 25880157 10.1038/srep09763 PMC5381748

[40] Granek JA, Gorbet DJ, Sergio LE. Extensive video-game experience alters cortical networks for complex visuomotor transformations. Cortex. 2010;46: 1165–1177. doi: 10.1016/j.cortex.2009.10.009 20060111 10.1016/j.cortex.2009.10.009

[41] Carey DP, Coleman RJ, Della Sala S. Magnetic misreaching. Cortex. 1997;33: 639–652. Available: http://www.ncbi.nlm.nih.gov/entrez/query.fcgi?cmd=Retrieve&db=PubMed&dopt=Citation&list_uids=9444466 9444466 10.1016/s0010-9452(08)70722-6

[42] Gorbet DJ, Sergio LE. Don’t watch where you’re going: The neural correlates of decoupling eye and arm movements. Behav Brain Res. 2016;298: 229–40. doi: 10.1016/j.bbr.2015.11.012 26589804 10.1016/j.bbr.2015.11.012

[43] Grigorova V, Bock O, Ilieva M, Schmitz G. Directional adaptation of reactive saccades and hand pointing movements is not independent. J Mot Behav. 2013;45: 101–6. doi: 10.1080/00222895.2012.750590 23441689 10.1080/00222895.2012.750590

[44] Neggers SF, Bekkering H. Gaze anchoring to a pointing target is present during the entire pointing movement and is driven by a non-visual signal. J Neurophysiol. 2000;83: 961–970. 10.1152/jn.2001.86.2.961 11495964

[45] Scherberger H, Andersen R a. Target selection signals for arm reaching in the posterior parietal cortex. J Neurosci. 2007;27: 2001–2012. doi: 10.1523/JNEUROSCI.4274-06.2007 17314296 10.1523/JNEUROSCI.4274-06.2007 PMC6673534

[46] Schubert T, Finke K, Redel P, Kluckow S, Muller H, Strobach T. Video game experience and its influence on visual attention parameters: An investigation using the framework of the Theory of Visual Attention (TVA). Acta Psychol (Amst). Elsevier B.V.; 2015;157: 200–214. doi: 10.1016/j.actpsy.2015.03.005 25834984 10.1016/j.actpsy.2015.03.005

[47] De Lisi R, Wolford JL. Improving children’s mental rotation accuracy with computer game playing. J Genet Psychol. 2002;163: 272–282. doi: 10.1080/00221320209598683 12230149 10.1080/00221320209598683

[48] Quaiser-Pohl C, Geiser C, Lehmann W. The relationship between computer-game preference, gender, and mental-rotation ability. Pers Individ Dif. 2006;40: 609–619. doi: 10.1016/j.paid.2005.07.015

[49] Okagaki L, Frensch PA. Effects of Video Game Playing on Measures of Spatial Performance : Gender Effects in Late Adolescence. J Appl Dev Psychol. 1994;15: 33–58.

[50] Subrahmanyam K, Greenfield PM. Effect of Video Game Practice on Spatial Skills in Girls and Boys. J Appl Dev Psychol. 1994;15: 13–32.

[51] Harwell D. More women play video games than boys, and other surprising facts lost in the mess of Gamergate. The Washington Post. 17 Oct 2014.

[52] Lucas K, Sherry JL. Sex Differences in Video Game Play. Communic Res. 2004;31: 499–523. doi: 10.1177/0093650204267930

[53] Thorson CM, Kelly JP, Forse RA, Turaga KK. Can we continue to ignore gender differences in performance on simulation trainers? J Laparoendosc Adv Surg Tech A. 2011;21: 329–33. doi: 10.1089/lap.2010.0368 21563940 10.1089/lap.2010.0368

[54] Kennedy AM, Boyle EM, Traynor O, Walsh T, Hill ADK. Video gaming enhances psychomotor skills but not visuospatial and perceptual abilities in surgical trainees. J Surg Educ. Elsevier Inc.; 2011;68: 414–420. doi: 10.1016/j.jsurg.2011.03.009 21821223 10.1016/j.jsurg.2011.03.009

[55] Fritz SL, Peters DM, Merlo AM, Donley J. Active video-gaming effects on balance and mobility in individuals with chronic stroke: a randomized controlled trial. Top Stroke Rehabil. 20: 218–25. doi: 10.1310/tsr2003-218 23841969 10.1310/tsr2003-218

[56] Levac DE, Miller P a. Integrating virtual reality video games into practice: clinicians’ experiences. Physiother Theory Pract. 2013;29: 504–12. doi: 10.3109/09593985.2012.762078 23362843 10.3109/09593985.2012.762078

[57] Clark JE, Lanphear AK, Riddick CC. The effects of video game playing on the response selection of elderly adults. J Gerontol. 1987;42: 82–85. 3794204 10.1093/geronj/42.1.82

[58] Dustman RE, Emmerson RY, Steinhaus LA, Shearer DE, Dustman TJ. The effects of videogame playing on neuropsychological performance of elderly individuals. J Gerontol. 1992;47: P168–71. Cited By (since 1996) 14\nExport Date 14 February 2012 1573200 10.1093/geronj/47.3.p168

[59] Basak C, Boot WR, Voss MW, Kramer AF. Can Training in a Real-Time Strategy Video Game Attenuate Cognitive Decline in Older Adults? Psychol Aging. American Psychological Association; 2008;23: 765–777. doi: 10.1037/A0013494 19140648 10.1037/a0013494 PMC4041116

[60] Gorbet DJ, Sergio LE. Preliminary sex differences in human cortical BOLD fMRI activity during the preparation of increasingly complex visually guided movements. Eur J Neurosci. 2007;25: 1228–1239. doi: 10.1111/j.1460-9568.2007.05358.x 17331218 10.1111/j.1460-9568.2007.05358.x

[61] Gorbet DJ, Mader LB, Richard Staines W. Sex-related differences in the hemispheric laterality of slow cortical potentials during the preparation of visually guided movements. Exp Brain Res. 2010;202: 633–646. doi: 10.1007/s00221-010-2170-1 20135101 10.1007/s00221-010-2170-1

[62] Gorbet DJ, Staines WR. Inhibition of contralateral premotor cortex delays visually guided reaching movements in men but not in women. Exp Brain Res. 2011;212: 315–325. doi: 10.1007/s00221-011-2731-y 21607701 10.1007/s00221-011-2731-y

[63] Boot WR, Blakely DP, Simons DJ. Do action video games improve perception and cognition? Front Psychol. 2011;2: 1–6. 21949513 10.3389/fpsyg.2011.00226 PMC3171788

[64] Oldfield RC. The assessment and analysis of handedness: the Edinburgh inventory. Neuropsychologia. 1971;9: 97–113. 5146491 10.1016/0028-3932(71)90067-4

[65] Chen Y, Monaco S, Byrne P, Yan X, Henriques DYP, Crawford JD. Allocentric versus egocentric representation of remembered reach targets in human cortex. J Neurosci. 2014;34: 12515–26. doi: 10.1523/JNEUROSCI.1445-14.2014 25209289 10.1523/JNEUROSCI.1445-14.2014 PMC6615499

[66] Galletti C, Gamberini M, Kutz DF, Fattori P, Luppino G, Matelli M. The cortical connections of area V6: an occipito-parietal network processing visual information. Eur J Neurosci. 2001;13: 1572–1588. 11328351 10.1046/j.0953-816x.2001.01538.x

[67] Passarelli L, Rosa MGP, Gamberini M, Bakola S, Burman KJ, Fattori P, et al Cortical connections of area V6Av in the macaque: a visual-input node to the eye/hand coordination system. J Neurosci. 2011;31: 1790–1801. doi: 10.1523/JNEUROSCI.4784-10.2011 21289189 10.1523/JNEUROSCI.4784-10.2011 PMC6623732

[68] de Jong BM, van der Graaf FH, Paans A. Brain activation related to the representations of external space and body scheme in visuomotor control. Neuroimage. 2001;14: 1128–35. doi: 10.1006/nimg.2001.0911 11697944 10.1006/nimg.2001.0911

[69] Medendorp WP, Goltz HC, Crawford JD, Vilis T. Integration of target and effector information in human posterior parietal cortex for the planning of action. JNeurophysiol. 2005;93: 954–962. doi: 10.1152/jn.00725.2004 15356184 10.1152/jn.00725.2004

[70] Monaco S, Chen Y, Medendorp WP, Crawford JD, Fiehler K, Henriques DYP. Functional magnetic resonance imaging adaptation reveals the cortical networks for processing grasp-relevant object properties. Cereb Cortex. 2014;24: 1540–1554. doi: 10.1093/cercor/bht006 23362111 10.1093/cercor/bht006

[71] Pitzalis S, Sdoia S, Bultrini A, Committeri G, Di Russo F, Fattori P, et al Selectivity to Translational Egomotion in Human Brain Motion Areas. PLoS One. 2013;8: 1–14. doi: 10.1371/journal.pone.0060241 23577096 10.1371/journal.pone.0060241 PMC3618224

[72] Vesia M, Crawford JD. Specialization of reach function in human posterior parietal cortex. Exp Brain Res. 2012;221: 1–18. doi: 10.1007/s00221-012-3158-9 22777102 10.1007/s00221-012-3158-9

[73] Fischer E, Bülthoff HH, Logothetis NK, Bartels A. Human Areas V3A and V6 Compensate for Self-Induced Planar Visual Motion. Neuron. 2012;73: 1228–1240. doi: 10.1016/j.neuron.2012.01.022 22445349 10.1016/j.neuron.2012.01.022

[74] Kravitz DJ, Saleem KS, Baker CI, Mishkin M. A new neural framework for visuospatial processing. Nat Rev Neurosci. Nature Publishing Group; 2011;12: 217–230. doi: 10.1167/11.11.923 10.1038/nrn3008 PMC3388718 21415848

[75] Lee H, Voss MW, Prakash RS, Boot WR, Vo LTK, Basak C, et al Videogame training strategy-induced change in brain function during a complex visuomotor task. Behav Brain Res. Elsevier B.V.; 2012;232: 348–357. doi: 10.1016/j.bbr.2012.03.043 22504276 10.1016/j.bbr.2012.03.043

[76] Miall RC, Reckess GZ, Imamizu H. The cerebellum coordinates eye and hand tracking movements. Nat Neurosci. 2001;4: 638–644. Available: http://www.ncbi.nlm.nih.gov/entrez/query.fcgi?cmd=Retrieve&db=PubMed&dopt=Citation&list_uids=11369946 doi: 10.1038/88465 11369946 10.1038/88465

[77] Striemer CL, Chouinard PA, Goodale MA, de Ribaupierre S. Overlapping neural circuits for visual attention and eye movements in the human cerebellum. Neuropsychologia. Elsevier; 2015;69: 9–21. doi: 10.1016/j.neuropsychologia.2015.01.024 25613405 10.1016/j.neuropsychologia.2015.01.024

[78] Balsters JH, Ramnani N. Cerebellar Plasticity and the Automation of First-Order Rules. J Neurosci. 2011;31: 2305–2312. doi: 10.1523/JNEUROSCI.4358-10.2011 21307266 10.1523/JNEUROSCI.4358-10.2011 PMC6633030

[79] Lacquaniti F, Perani D, Guigon E, Bettinardi V, Carrozzo M, Grassi F, et al Visuomotor transformations for reaching to memorized targets: a PET study. Neuroimage. 1997;5: 129–146. doi: 10.1006/nimg.1996.0254 9345543 10.1006/nimg.1996.0254

[80] Brown GG, Caligiuri M, Meloy MJ, Eberson SC, Kindermann SS, Frank LR, et al Functional brain asymmetries during visuomotor tracking. J Clin Exp Neuropsychol. 2004;26: 356–368. doi: 10.1080/13803390490510086 15512926 10.1080/13803390490510086

[81] Sinanaj I, Cojan Y, Vuilleumier P. Inter-individual variability in metacognitive ability for visuomotor performance and underlying brain structures. Conscious Cogn. Elsevier Inc.; 2015;36: 327–337. doi: 10.1016/j.concog.2015.07.012 26241023 10.1016/j.concog.2015.07.012

[82] Bernardi G, Ricciardi E, Sani L, Gaglianese A, Papasogli A, Ceccarelli R, et al How Skill Expertise Shapes the Brain Functional Architecture: An fMRI Study of Visuo-Spatial and Motor Processing in Professional Racing-Car and Naive Drivers. PLoS One. 2013;8: 1–11. doi: 10.1371/journal.pone.0077764 24204955 10.1371/journal.pone.0077764 PMC3799613

[83] Gobel EW, Parrish TB, Reber PJ. Neural correlates of skill acquisition: Decreased cortical activity during a serial interception sequence learning task. Neuroimage. Elsevier Inc.; 2011;58: 1150–1157. doi: 10.1016/j.neuroimage.2011.06.090 21771663 10.1016/j.neuroimage.2011.06.090 PMC3171628

[84] Haslinger B, Erhard P, Altenmuller E, Hennenlotter A, Schwaiger M, Grafin von Einsiedel H, et al Reduced recruitment of motor association areas during bimanual coordination in concert pianists. Hum Brain Mapp. Wiley-Liss, Inc; 2004;22: 206–215. doi: 10.1002/hbm.20028 15195287 10.1002/hbm.20028 PMC6871883

[85] Jancke L, Shah NJ, Peters M. Cortical activations in primary and secondary motor areas for complex bimanual movements in professional pianists. Cogn Brain Res. 2000;10: 177–183. doi: 10.1016/S0926-6410(00)00028-8 10.1016/s0926-6410(00)00028-8 10978706

[86] Krings T, Topper R, Foltys H, Erberich S, Sparing R, Willmes K, et al Cortical activation patterns during complex motor tasks in piano players and control subjects. A functional magnetic resonance imaging study. Neurosci Lett. 2000;278: 189–193. Available: http://www.ncbi.nlm.nih.gov/entrez/query.fcgi?cmd=Retrieve&db=PubMed&dopt=Citation&list_uids=10653025 10653025 10.1016/s0304-3940(99)00930-1

[87] Morris MC, Frodl T, D’Souza A, Fagan AJ, Ridgway PF. Assessment of competence in surgical skills using functional magnetic resonance imaging: A feasibility study. J Surg Educ. Elsevier; 2015;72: 198–204. doi: 10.1016/j.jsurg.2014.09.007 25439177 10.1016/j.jsurg.2014.09.007

[88] Petrini K, Pollick FE, Dahl S, McAleer P, McKay L, Rocchesso D, et al Action expertise reduces brain activity for audiovisual matching actions: An fMRI study with expert drummers. Neuroimage. 2011;56: 1480–1492. doi: 10.1016/j.neuroimage.2011.03.009 21397699 10.1016/j.neuroimage.2011.03.009

[89] Culham JC, Danckert SL, DeSouza JFX, Gati JS, Menon RS, Goodale M a. Visually guided grasping produces fMRI activation in dorsal but not ventral stream brain areas. Exp Brain Res. 2003;153: 180–189. doi: 10.1007/s00221-003-1591-5 12961051 10.1007/s00221-003-1591-5

[90] Castel AD, Pratt J, Drummond E. The effects of action video game experience on the time course of inhibition of return and the efficiency of visual search. Acta Psychol (Amst). 2005;119: 217–230. doi: 10.1016/j.actpsy.2005.02.004 15877981 10.1016/j.actpsy.2005.02.004

[91] Dye MWG, Green CS, Bavelier D. The development of attention skills in action video game players. Neuropsychologia 2009;47: 1780–1789. doi: 10.1016/j.neuropsychologia.2009.02.002 19428410 10.1016/j.neuropsychologia.2009.02.002 PMC2680769

[92] Barrett KM, Brott TG, Brown RD, Frankel MR, Worrall BB, Silliman SL, et al Sex differences in stroke severity, symptoms, and deficits after first-ever ischemic stroke. J Stroke Cerebrovasc Dis. 2007;16: 34–9. doi: 10.1016/j.jstrokecerebrovasdis.2006.11.002 17689390 10.1016/j.jstrokecerebrovasdis.2006.11.002 PMC1945157

[93] Di Carlo A, Lamassa M, Baldereschi M, Pracucci G, Basile AM, Wolfe CD a, et al Sex differences in the clinical presentation, resource use, and 3-month outcome of acute stroke in Europe: Data from a multicenter multinational hospital-based registry. Stroke. 2003;34: 1114–1119. doi: 10.1161/01.STR.0000068410.07397.D7 12690218 10.1161/01.STR.0000068410.07397.D7

[94] Dillard C, Ditchman N, Nersessova K, Foster N, Wehman P, West M, et al Post-concussion symptoms in mild traumatic brain injury: findings from a paediatric outpatient clinic. Disabil Rehabil. 2016;8288: 1–7. doi: 10.3109/09638288.2016.1152602 26971917 10.3109/09638288.2016.1152602

[95] Haaxma CA, Bloem BR, Borm GF, Oyen WJ, Leenders KL, Eshuis S, et al Gender differences in Parkinson’s disease. J Neurol Neurosurg Psychiatry. 2007;78: 819–824. doi: 10.1136/jnnp.2006.103788 17098842 10.1136/jnnp.2006.103788 PMC2117736

[96] Labiche LA. Chan W. Saldin KR. Morgenstern L. Sex and acute stroke. Ann Emerg Med. 2002;40: 453–460. 12399786 10.1067/mem.2002.128682

[97] Liu M, Dziennis S, Hurn PD, Alkayed NJ. Mechanisms of gender-linked ischemic brain injury. Restor Neurol Neurosci. 2009;27: 163–179. doi: 10.3233/RNN-2009-0467 19531872 10.3233/RNN-2009-0467 PMC2826890

[98] Wooten GF, Currie LJ, Bovbjerg VE, Lee JK, Patrie J. Are men at greater risk for Parkinson’s disease than women? J Neurol Neurosurg Psychiatry. 2004;75: 637–639. doi: 10.1136/jnnp.2003.020982 15026515 10.1136/jnnp.2003.020982 PMC1739032

